# Software Sensor to Enhance Online Parametric Identification for Nonlinear Closed-Loop Systems for Robotic Applications

**DOI:** 10.3390/s21113653

**Published:** 2021-05-24

**Authors:** Lilia Sidhom, Ines Chihi, Ernest Nlandu Kamavuako

**Affiliations:** 1 Laboratory of Energy Applications and Renewable Energy Efficiency (LAPER), El Manar University, Tunis 1068, Tunisia; lilia.sidhom@enib.rnu.tn; 2 National Engineering School of Bizerta, Carthage University, Tunis 7080, Tunisia; 3 Département Ingénierie, Campus Kirchberg, Faculté des Sciences, des Technologies et de Médecine, Université du Luxembourg, 1359 Luxembourg, Luxembourg; 4 Department of Engineering, King’s College London, London WC2R 2LS, UK; ernest.kamavuako@kcl.ac.uk

**Keywords:** identification, dynamic sliding mode, direct and cross-validation, robot application

## Abstract

This paper proposes an online direct closed-loop identification method based on a new dynamic sliding mode technique for robotic applications. The estimated parameters are obtained by minimizing the prediction error with respect to the vector of unknown parameters. The estimation step requires knowledge of the actual input and output of the system, as well as the successive estimate of the output derivatives. Therefore, a special robust differentiator based on higher-order sliding modes with a dynamic gain is defined. A proof of convergence is given for the robust differentiator. The dynamic parameters are estimated using the recursive least squares algorithm by the solution of a system model that is obtained from sampled positions along the closed-loop trajectory. An experimental validation is given for a 2 Degrees Of Freedom (2-DOF) robot manipulator, where direct and cross-validations are carried out. A comparative analysis is detailed to evaluate the algorithm’s effectiveness and reliability. Its performance is demonstrated by a better-quality torque prediction compared to other differentiators recently proposed in the literature. The experimental results highlight that the differentiator design strongly influences the online parametric identification and, thus, the prediction of system input variables.

## 1. Introduction

The general problem of manipulators still lies in the large number of their physical parameters, which are usually not well known. To correctly build a mathematical model of such systems, different parameter identification techniques exist, which can be divided into two categories: direct and indirect approaches [1]. For the latter approach, the controller expression is related to the definition of the identification algorithm, whereas direct methods can identify the parameters of the system model independently of the structure controller applied to the robot. This research study relates to direct methods.

Most of the identification methods defined in the literature use the direct model of the system [1,2]. The formulation of the identification problem with such a model can lead to nonlinear optimization. Unfortunately, such a solution suffers from various difficulties, such as having multiple feasible regions; each one has multiple locally optimal points, specifically when the objective function or any of the constraints is non-convex. This non-convexity issue is more problematic for nonlinear models due to the methods that give coherent initialization steps. These methods may not be available for many nonlinear model structures. Other methods exist in the literature which are based on neural networks [3,4,5,6]. Identification methods based on nonlinear observers/filters such as methods based on the extended Kalman filter [7], high-gain observers [8], and observers based on sliding modes [9,10,11,12] have also been proposed. The drawback of these last ones is that they depend on a priori knowledge of the modeled system, which can sometimes be quite complex and inaccurate. Nevertheless, the direct model for mechanical systems is often flat, thus promoting the use of the inverse model that can be considered linear with respect to a set of dynamic parameters. By relying on the inverse model, the problem of identification is reduced to a minimization of a prediction error of unknown parameters. This minimization is performed according to a chosen criterion that is generally of a quadratic form. Thus, the vector of unknown parameters can be obtained through optimization based on different models that exist in the literature. Some of these models include the least-squares (LS) method [13], the weighted least squares (WLS) method [7], the maximum likelihood estimation method [14], and the recursive least squares (RLS) algorithm. The RLS algorithm has a major advantage in that it requires multiple iterations, making online identification easier. It has good convergence and provides for small estimation errors in the stationary case and the underlying normally distributed noise [15]. To further improve the performance of this algorithm, a forgetting factor (FF) term can be included [15,16]. Apart from the possibility of application of a classical algorithm such as the RLS, the use of the inverse model requires also a good prior estimation of the state and its derivatives. Therefore, both filtering and differentiation algorithm methods play a key role in such identification processes. Only a few studies have investigated the procedure of online parametric identification by associating a differentiator and have implemented it for fear of amplifying the measurement noise. The measurement noise properties are not known beforehand, especially in practice. Thus, the main challenge here is to find a suitable online algorithm that can guarantee a good compromise between the differentiation accuracy and noise rejection. This algorithm is defined as a soft sensor to estimate the successive derivatives of the system state and also to replace a real physical sensor. The use of such a sensor makes it possible to reduce the number of speed and acceleration sensors. Indeed, if we have an n-DoF manipulator, we need 2 × n physical sensors. In addition, a software sensor rarely breaks down, never wears out, and does not require calibration. Soft sensors have become increasingly important in various applications, such as the design of controllers, observers [17,18], the sensorless control approach, and diagnostic problems [19]. A limited number of studies have proposed software sensors based on the differentiation algorithm [20,21]. In [21], the authors addressed the problem of online identification for uncertain nonlinear systems based on the state derivative estimation method. For their purpose, they have approximated the system model online, using a neural network with some feedback term to compensate for the modeling errors and exogenous disturbances. To achieve the identification process, a comparative study of different differentiation algorithms—including the high gain observer, Levant’s first-order sliding mode differentiator, backward difference, and central difference methods—was performed. This study was done with simulation tests, and only a first-order state derivative was computed. In [22,23,24], the authors proposed the parameter identification of a robot manipulator, using a causal Jacobi orthogonal-based algebraic differentiator to compute the joint acceleration from the position measurements. The principle of this kind of differentiator, as proposed by [25], is based on the truncated series of the Taylor expansion signal to be estimated. Although such an algorithm allows efficient attenuation of the noise, it is sensitive to the truncation order, to the size of the sliding window estimation, and especially to the setting of its parameters. All this makes it difficult to set these parameters in order to obtain a good estimate. An alternative differentiator that is based on a higher-order sliding mode can be used. For the state estimation, a robust differentiator based on the sliding mode technique is applied, as in [26,27,28]. In fact, the author uses the well-known Levant’s differentiator, the so-called Super Twisting (ST) algorithm [27], and the LS method for online parametric identification of nonlinear systems in the presence of noise. Different new forms of the popular first-order ST differentiator have been proposed and applied with satisfactory results [26,27,28,29,30,31,32,33,34].

In this paper, we propose a soft sensor based on the sliding mode differentiation algorithm to enhance an online dynamic parameter identification procedure for a robot manipulator based on its inverse model. The software sensor outputs are, therefore, injected into an RLS estimator to reach this aim. To the best of our knowledge, only a few software sensors based on the second-order adaptive sliding mode algorithm allow velocity and acceleration to be provided simultaneously [35]. The main features of the proposed online identification approach may be summarized as follows: (1) Estimation of both the velocity and acceleration of each joint by the proposed software sensor, which is robust with respect to the noisy data, without any knowledge of the statistical properties of the noise. (2) A comparative analysis allows assessing the performance of the proposed identification approach with respect to other software sensor-based differentiators. (3) The proposed approach allows identifying model parameters with very good performances. This paper is organized as follows. Section 2 outlines the general principle of the proposed method. Section 3 presents the system application and the different implementation steps of the identification process. Section 4 is dedicated to the experimental results, validation, and discussion.

## 2. Manipulator Modeling and Parameter Identification

This paper considers the parameter identification problem of closed-loop nonlinear systems [36]. In fact, the problem is especially interesting for open-loop unstable systems and also for operating safety reasons. The correlation between the input/noise in the closed-loop technique is the major area in which it differs from the open-loop methods. Considering some input/output constraints, it is possible to consider that the performances of the open-loop and closed-loop identification techniques are the same [37,38]. For both techniques, the manipulator input-output signals are recorded as the system tracks some pre-defined trajectories. Thus, the main practical challenge for the identification procedure is the noises that could be provided by the data acquisition chain, sensors, and even the process. The defined closed-loop online identification approach can be applied to any robot manipulator no matter how many inputs/outputs it has. The only condition is that the direct model must be flat [39]. Therefore, exploiting the properties of the inverse model gives us a linear model with respect to a grouping of physical parameters.

**Figure 1:**
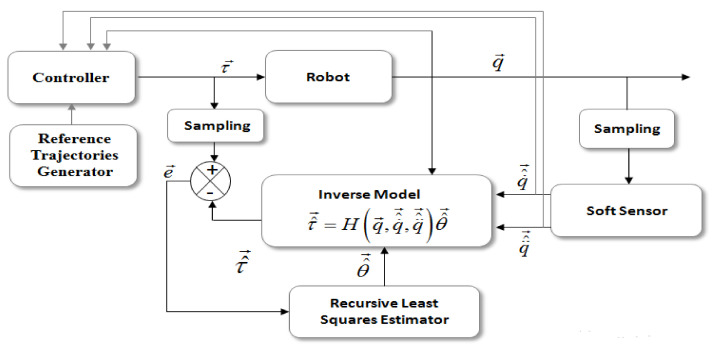
The general principle of the closed-loop identification approach.

The problem, then, becomes a minimization problem of a prediction error $\vec{e}$ with respect to the vector of unknown parameters $\vec{\theta }$ obtained by an RLS algorithm. The overall identification procedure is illustrated in Figure 1. The prediction error $\vec{e}$ represents the difference between the system inputs $\vec{\tau }$ and their estimates $\vec{\hat{\tau }}$. All mechanical quantities denoted by the vectors $\vec{\tau }$, $\vec{q}$, $\vec{\hat{\tau }}$, $\vec{\hat{\dot{q}}}$, $\vec{\hat{\ddot{q}}}$, and $\vec{\hat{\theta }}$ are the vector of system inputs, outputs, input estimation, velocity estimation, acceleration estimation, and the vector parameter estimation, respectively. This approach requires in return a good prior estimate of the state derivatives. Thus, the software sensor that must be chosen plays a key role in the identification process. For such sensors, the first issue concerns the noises on their outputs and the second issue is about the estimation accuracy of the velocities and accelerations. For the closed-loop identification approach, the robots are usually position-controlled. Different basic controllers are generally used such as PD and PID controllers. Although these controllers do not have good precision compared with others, they are widely used for identification due to their ease of adjustment. Commonly, all the electrical parts relating to the actuators are neglected because their dynamics are very fast compared to the mechanical parts. Thus, the relationship between the torque and the current signal provided from the electrical motors is modeled by a static gain. The value of this gain is known a priori from the manufacturer data.

### 2.1. Dynamic Model

This section presents the class of nonlinear systems to be identified. Let us define a mathematical dynamic model of an n-link rigid robot in joint space as follows:

(1) $$A(q)\ddot{q}+C\left(q,\dot{q}\right)\dot{q}+g(q)+\tau_{F}=\tau$$

where $q\left(t\right)={\left[q_{i}\left(t\right)\right]}^{T},\dot{q}\left(t\right)={\left[{\dot{q}}_{i}\left(t\right)\right]}^{T}$ and $\ddot{q}\left(t\right)={\left[{\ddot{q}}_{i}\left(t\right)\right]}^{T}$ are the vector of joint position, the joint velocity, and the acceleration, respectively. $A\left(q\right)$ is the inertia matrix of the robot, $C\left(q,\dot{q}\right)$ is the matrix containing the centrifugal, and the Coriolis torques, $g(q)\in {\mathbb{R}}^{n}$ is the vector of the gravitational vector $\tau_{F}$ and τ, are the friction forces and the joint torque vectors, respectively. The friction forces vector is defined by the dry friction and viscous friction terms as follows:

(2) $$\tau_{F}=F_{v}\dot{q}+F_{s}sign\left(\dot{q}\right)$$

where $F_{v},F_{s}\in {\mathbb{R}}^{\left(n*n\right)}$ are constant diagonal matrices representing viscous and Coulomb friction parameters, respectively. Let us rewrite Equation (1) in a linearly parameterized form with respect to a vector of $n_{\theta }$ dynamic parameters [40]:

(3) $$\tau =H\left(q,\dot{q},\ddot{q}\right)\theta$$

where $H\left(q,\dot{q},\ddot{q}\right)\in {\mathbb{R}}^{\left(n*n_{\theta }\right)}$ is a regression matrix and $\theta \in {\mathbb{R}}^{\left(n_{\theta }*1\right)}$ is a vector of the parameter model that represents the minimal set of identifiable parameters to describe the dynamic model. The vector θ is obtained by regrouping some of the base parameters with respect to the QR decomposition [39] or via some linear relations [2,7].

### 2.2. Algorithm for the Proposed Soft Sensor

The main advantage of higher-order sliding mode (HOSM) algorithms is the ease of their implementation in real-time, which justifies their successful applications [41,42,43]. However, their major drawback is the gain setting in real-time. In fact, the gain setting requires that the Lipschitz constant of the derivative signal, which is difficult to know in practice since the signal to estimate is not necessarily known in advance, must be accurately known beforehand. Thus, for online applications, it is necessary to adjust the gains each time the basic signal changes. Therefore, the major difficulty lies in the gain selection of such differentiators. To fix this problem, various new sliding mode differentiators have been proposed in the literature, where the aim is to define an adaptive form of this classic scheme.

Let the input signal $g\left(t\right)$ be a function defined on $[0,\infty [$ and have a Lipschitz derivative *C* defined as: $\forall t\in [0,\infty [;\max\left(\left|\frac{d^{\left(n+1\right)}}{dt^{\left(n+1\right)}}g\left(t\right)\right|\right)\leq C$, where *C* is unknown. This input signal can be defined as follows:

(4) $$g(t)=g_{0}(t)+\xi (t)$$

where ${g_{0}}^{\left(t\right)}$ is an unknown base signal with the (*n* + 1)th derivative having some Lipschitz constant C>0 and ξ(t) is a bounded Lebesgue-measurable noise with unknown features; it is defined by: $\left|\xi (t)\right|<\varepsilon$, where ε is sufficiently small.

The basic form of the first-order differentiator ST and the nth-order differentiator are defined in [27,28], respectively. The ST algorithm is described by Equation (5). By referring to [26], a basic form of the second-order sliding mode differentiator (2SMD) (for n=2) is defined by Equation (6):

(5) $$\left\{\begin{array}{l} {\dot{z}}_{0}=y_{0}\\ y_{0}=-\eta_{0}{\left|z_{0}-g\right|}^{\frac{1}{2}}sign\left(z_{0}-g\right)+z_{1}\\ {\dot{z}}_{1}=-\eta_{1}sign\left(z_{1}-y_{0}\right) \end{array}\right.$$

(6) $$\left\{\begin{array}{l} {\dot{z}}_{0}=y_{0}\\ y_{0}=-\eta_{0}{\left|z_{0}-g\right|}^{\frac{3}{2}}sign\left(z_{0}-g\right)+z_{1}\\ {\dot{z}}_{1}=y_{1} \end{array}\right.\;,\;\;\left\{\begin{array}{l} y_{1}=-\eta_{1}{\left|z_{1}-y_{0}\right|}^{\frac{1}{2}}sign\left(z_{1}-y_{0}\right)+z_{2}\\ {\dot{z}}_{2}=-\eta_{2}sign\left(z_{2}-y_{1}\right)=-\eta_{2}sign\left(z_{1}-y_{0}\right) \end{array}\right.$$

where $\eta_{i},i=\left\{0,\ldots ,n\right\}$ are differentiator parameters, which are positive, depending on the Lipschitz constant *C* of $g_{0}^{n+1}(t)$, and *y*_0_ and *y*_1_ are the differentiator outputs.

At time *t* = 0, these differentiators can well be performed as follows: $z_{0}\left(0\right)=g\left(0\right),z_{i}\left(0\right)=0,i\in \left\{1,2\right\}$ after finite-time convergence and in noiseless cases, $z_{1}=y_{0}$ is the estimation of ${\dot{g}}_{0}(t)$, and $z_{2}=y_{1}$ is the estimation of ${\ddot{g}}_{0}(t).$ In the equation systems (5) and (6), the quantities that present the *sign(.)* functions must theoretically vanish in finite time, but it is impossible to achieve this due to different inaccuracy sources as the measurement errors. In addition, this problem is amplified by the presence of discontinuities, which come from the *sign(.)* functions, in these equations. These latter produce the so-called chattering effect on the estimated signals. To overcome this problem, it is necessary to have adequate values of the differentiator parameters to also have good accuracy and minimize the chattering effect as much as possible. In some previous works [44], it was possible to replace the “*sign (.)”* with the “*sat(.)”* function; this makes it possible to slightly reduce the noise amplification in spite of the convergence algorithm, which can occur in the event of an inadequate slope value.

To define an algorithm with a compromise between the exactness and the level of noise for the considered signal, the new scheme of 2SMD is proposed. This solution permits some dynamic laws on the estimator’s settings that will be exposed by the following.

Let us define the proposed second-order sliding mode differentiator (P2SMD) as:

(7) $$\left\{\begin{array}{l} {\dot{z}}_{0}=y_{0};\\ y_{0}=-{\hat{\eta }}_{0}{\left|e_{0}\right|}^{\frac{2}{3}}sign(e_{0})+z_{1}-\Gamma_{0}e_{0} \end{array}\right.\;,\;\left\{\begin{array}{l} {\dot{z}}_{1}=y_{1};\\ y_{1}=-{\hat{\eta }}_{1}{\left|e_{1}\right|}^{\frac{1}{2}}sign(e_{1})-{\hat{\lambda }}_{2}\int_{0}^{t}sign\left(e_{1}\right)dt-\Gamma_{1}e_{1} \end{array}\right.$$

where ${\hat{\eta }}_{0},{\hat{\eta }}_{1},{\hat{\eta }}_{2}$ are dynamic gains, $\Gamma_{0}$, $\Gamma_{1}$ are convergence gains, and *e*_0_, *e*_1_ are the sliding functions, which are defined as:

(8) $$e_{0}=z_{0}-g\;\;,\;e_{1}=z_{1}-y_{0}$$

The dynamic gains ${\hat{\eta }}_{i},i\in \left\{0,1,2\right\}$ are defined as:

(9) $$\left\{\begin{array}{l} {\dot{\hat{\eta }}}_{0}=\left[{\left|e_{0}\right|}^{\frac{2}{3}}sign\left(e_{0}\right)\right]e_{0},\;\;\;{\hat{\eta }}_{0}\left(0\right)\geq 0\;\;\;and\;\;\;{\dot{\hat{\eta }}}_{0}>0\;\;\;\forall t>0\\ {\dot{\hat{\eta }}}_{1}=\left[{\left|e_{1}\right|}^{\frac{1}{2}}sign\left(e_{1}\right)\right]e_{1},\;\;\;{\hat{\eta }}_{1}\left(0\right)\geq 0\;\;\;and\;\;\;{\dot{\hat{\eta }}}_{1}>0\;\;\;\forall t>0\\ {\dot{\hat{\eta }}}_{2}=e_{1}\int_{0}^{t}sign\left(e_{1}\right)dt \end{array}\right.$$

**Theorem** **1.** *For $\Gamma_{0},\Gamma_{1}>0$ and with the dynamic gains ${\hat{\eta }}_{i},i\in \left\{0,1,2\right\}$ defined by system Equation (9), the system trajectories (7) converge locally and asymptotically towards the equilibrium point $e_{0}=e_{1}=0$, with the following assumption:*

(10) $$\dot{g}\left(t\right)=-\eta_{0}^{*}{\left|e_{0}\right|}^{\frac{2}{3}}sign\left(e_{0}\right)+z_{1},\;\;\ddot{g}\left(t\right)=-\eta_{1}^{*}{\left|e_{1}\right|}^{\frac{1}{2}}sign\left(e_{1}\right)-\lambda_{2}^{*}\int_{0}^{t}sign\left(e_{1}\right)dt$$

*where $\eta_{0}^{*},\eta_{1}^{*},\eta_{2}^{*}$ are positive constants that are unknown a priori.*

**Proof** **of** **Theorem** **1.** Let $\sigma_{0}=e_{0}-g$. With this new coordinate, the first two equations of the system (7) can be re-written as follows:

(11) $${\dot{\sigma }}_{0}=-{\hat{\eta }}_{0}{\left|\sigma_{0}\right|}^{\frac{2}{3}}sign\left(\sigma_{0}\right)-\Gamma_{0}\sigma_{0}+\sigma_{1}$$

where $\sigma_{1}=z_{1}-\dot{g}$, ${\hat{\eta }}_{0}\geq 0$, and $\Gamma_{0}>0$. From Equation (11) we have: $\sigma_{1}-{\dot{\sigma }}_{0}=sign\left(\sigma_{0}\right)\left[{\hat{\eta }}_{0}{\left|\sigma_{0}\right|}^{\frac{2}{3}}+\Gamma_{0}\left|\sigma_{0}\right|\right]$. Then, we have:

(12) $$sign\left(\sigma_{1}-{\dot{\sigma }}_{0}\right)=sign\left(\sigma_{0}\right)$$

Subtracting $\dot{g}\left(t\right)$ on both sides of the second Equation of (7), we obtain:

(13) $$y_{0}-\dot{g}\left(t\right)=-{\hat{\eta }}_{0}{\left|\sigma_{0}\right|}^{\frac{2}{3}}sign\left(\sigma_{0}\right)-\Gamma_{0}\sigma_{0}+z_{1}-\dot{g}\left(t\right)$$

Substituting $\dot{g}\left(t\right)$ in Equation (13) with its expression in Equation (10), we obtain the following equation:

(14) $${\dot{\sigma }}_{0}=-{\tilde{\eta }}_{0}{\left|\sigma_{0}\right|}^{\frac{2}{3}}sign\left(\sigma_{0}\right)-\Gamma_{0}\sigma_{0}$$

with ${\tilde{\eta }}_{0}={\hat{\eta }}_{0}-\eta_{0}^{*}$, which is an error between the estimated gain value and the gain value known a priori. Considering now that $\sigma_{1}=z_{1}-\dot{g}$, it gives $s_{1}=\sigma_{1}-{\dot{\sigma }}_{0}$. Subtracting $\ddot{g}\left(t\right)$ from both sides of the last equation of (7), we have:

(15) $${\dot{\sigma }}_{1}=-{\tilde{\eta }}_{1}{\left|\sigma_{1}-{\dot{\sigma }}_{0}\right|}^{\frac{1}{2}}sign\left(\sigma_{1}-{\dot{\sigma }}_{0}\right)-\Gamma_{0}\left(\sigma_{1}-{\dot{\sigma }}_{0}\right)-{\tilde{\eta }}_{2}\int_{0}^{t}sign\left(\sigma_{1}-{\dot{\sigma }}_{0}\right)dt$$

Let us define a Lyapunov function as:

(16) $$V(\sigma_{0},\sigma_{1},{\tilde{\eta }}_{i})=\frac{1}{2}\sigma_{0}^{2}+\frac{1}{2}{\left(\sigma_{1}-{\dot{\sigma }}_{0}\right)}^{2}+\frac{1}{2}\sum_{i=0}^{2}{\tilde{\eta }}_{i}^{2},\;\;i\in \left\{0,1,2\right\}$$

Let us define the equilibrium point such as $X_{eq}={\left(0,0,0\right)}^{T}$. Then, the derivative of the Lyapunov function defined by Equation (16) is given by:

(17) $$\dot{V}=\sigma_{0}{\dot{\sigma }}_{0}+\left(\sigma_{1}-{\dot{\sigma }}_{0}\right)\left({\dot{\sigma }}_{1}-{\ddot{\sigma }}_{0}\right)+{\tilde{\eta }}_{0}{\dot{\hat{\eta }}}_{0}+{\tilde{\eta }}_{1}{\dot{\hat{\eta }}}_{1}+{\tilde{\eta }}_{2}{\dot{\hat{\eta }}}_{2}$$

with $\sigma_{0}{\dot{\sigma }}_{0}+{\tilde{\eta }}_{0}{\dot{\hat{\eta }}}_{0}=-\Gamma_{0}\sigma_{0}^{2}$, and we can also obtain:

(18) $$\dot{V}=-\Gamma_{0}\sigma_{0}^{2}-\Gamma_{1}{\left(\sigma_{1}-{\dot{\sigma }}_{0}\right)}^{2}-\left(\sigma_{1}-{\dot{\sigma }}_{0}\right){\ddot{\sigma }}_{0}$$

We have:

(19) $$-\left(\sigma_{1}-{\dot{\sigma }}_{0}\right){\ddot{\sigma }}_{0}=-\left(\sigma_{1}-{\dot{\sigma }}_{0}\right)\left[-{\dot{\hat{\eta }}}_{0}\right.{\left|\sigma_{0}\right|}^{\frac{2}{3}}sign\left(\sigma_{0}\right)\left.-\Gamma_{0}{\dot{\sigma }}_{0}-\frac{2}{3}{\tilde{\eta }}_{0}{\left|\sigma_{0}\right|}^{-\frac{1}{3}}{\dot{\sigma }}_{0}\right]$$

Replacing ${\hat{\eta }}_{0}$ (see system (9)) in Equation (19) by its expression, the following equality is satisfied:

(20) $$-\left(\sigma_{1}-{\dot{\sigma }}_{0}\right){\ddot{\sigma }}_{0}=-\left|\sigma_{1}-{\dot{\sigma }}_{0}\right|\left[-{\left|\sigma_{0}\right|}^{\frac{7}{3}}+\Gamma_{0}^{2}\left|\sigma_{0}\right|\right.\left.+\frac{2}{3}{\tilde{\eta }}_{0}^{2}{\left|\sigma_{0}\right|}^{\frac{1}{3}}+\frac{5}{3}{\tilde{\eta }}_{0}\Gamma_{0}{\left|\sigma_{0}\right|}^{\frac{2}{3}}\right]$$

Consequently, Equation (17) can be rewritten as follows:

(21) $$\dot{V}=-\Gamma_{0}\sigma_{0}^{2}-\Gamma_{1}{\left(\sigma_{1}-{\dot{\sigma }}_{0}\right)}^{2}-\frac{2}{3}\left|\sigma_{1}-{\dot{\sigma }}_{0}\right|{\tilde{\eta }}_{0}^{2}{\left|\sigma_{0}\right|}^{\frac{1}{3}}-\left|\sigma_{1}-{\dot{\sigma }}_{0}\right|\left[-{\left|\sigma_{0}\right|}^{\frac{7}{3}}+\frac{5}{3}{\tilde{\eta }}_{0}\Gamma_{0}{\left|\sigma_{0}\right|}^{\frac{2}{3}}+\Gamma_{0}^{2}\left|\sigma_{0}\right|\right]$$

To show that $\dot{V}$ is negative, it is sufficient to prove that:

(22) $$Ζ=\left[-{\left|\sigma_{0}\right|}^{\frac{7}{3}}+\frac{5}{3}{\tilde{\eta }}_{0}\Gamma_{0}{\left|\sigma_{0}\right|}^{\frac{2}{3}}+\Gamma_{0}^{2}\left|\sigma_{0}\right|\right]\geq 0$$

Therefore, let us assume that $\left|{\tilde{\eta }}_{0}\right|\leq {\tilde{\eta }}_{0M}$, where ${\tilde{\eta }}_{0M}$ is a positive constant satisfying the following inequality:

(23) $${\tilde{\eta }}_{0M}<\frac{3}{5}\Gamma_{0}{\left|\sigma_{0}\right|}^{\frac{1}{3}}$$

To obtain the condition defined by Equation (23), one must choose $\Gamma_{0}$ such that:

(24) $$\Gamma_{0}\left[{\tilde{\eta }}_{0}+\frac{3}{5}\Gamma_{0}{\left|\sigma_{0}\right|}^{\frac{1}{3}}\right]\geq \frac{3}{5}{\left|\sigma_{0}\right|}^{\frac{5}{3}}$$

It should be noted that the only condition to satisfy inequalities (24) and (25) is to have a positive value of $\Gamma_{0}$ (namely a high value). □

**Remark** **1.** *It can be noticed that $\dot{V}$ is a negative function $\forall (\sigma_{0},\sigma_{1},{\tilde{\eta }}_{i})\in {\mathbb{R}}^{3}$ and it cancels when ${\left(\sigma_{0}=0,\sigma_{1}=0,{\tilde{\eta }}_{i}\right)}^{T}\neq X_{eq}$. Therefore, $\dot{V}$ is a globally semi-negative definite function on ${\mathbb{R}}^{3}$ and it is defined as a locally negative definite function of ${\mathbb{R}}^{3}{\left|\left(\sigma_{0}=0,\sigma_{1}=0,{\tilde{\eta }}_{i}\right)\right.}^{T}$. Consequently, with the defined Lyapunov function, a global convergence of ${\mathbb{R}}^{3}$ of the equilibrium point is proved. And a local asymptotic convergence of the algorithm has also been proven on ${\mathbb{R}}^{3}{\left|\left(\sigma_{0}=0,\sigma_{1}=0,{\tilde{\eta }}_{i}\right)\right.}^{T}$. The global asymptotic convergence of the algorithm could not be demonstrated using the LaSalle’s invariance principle.*

**Remark** **2.** *The convergence of the dynamic gains $\eta_{0}^{*}$ and $\eta_{1}^{*}$ is not guaranteed. As opposed to this, these dynamic gains change over time in a continuous way according to the imposed adaptation laws. Depending on the initial values of the differentiator gains, the dynamic gains have a bounded evolution for all simulation tests that are carried out.*

**Remark** **3.** *The behavior of the P2SMD is equivalent to the behavior of a bandpass filter for small values of a couple of gains $\left(\Gamma_{0},\Gamma_{1}\right)$. In fact, the setting $\left(\Gamma_{0},\Gamma_{1}\right)$ is specified for the two possible cases: with noiseless signals and with a noisy signal. For the first case, if the gain values $\left(\Gamma_{0},\Gamma_{1}\right)$ become high, then the convergence time of the algorithms becomes quick. For the second case, there is some compromise between the convergence time and the noise amplification rate. In fact, the linear terms $\Gamma_{i}s_{i}i\in \left\{0,1\right\}$ defined by the $\left(\Gamma_{0},\Gamma_{1}\right)$ gains are the key to smoother output differentiators compared to the basic scheme. Then, it is necessary not to choose values that are too high.*

### 2.3. Recursive Least Squares Estimator

To track the imposed reference trajectories that excite the system dynamics of the robot manipulator, the inputs/outputs of this last one are sampled. For our case, a recursive least squares (RLS) estimation method is used. Then, via the measurement of torques and positions of each joint, the root-mean-square residual error of the model is optimized. This error is the difference between the signal torque and its estimated value. Thereafter, a cost function resting on this error is used to obtain a parameter identification formula under the assumption that the measurement errors are negligible. The joint positions/torques are measured with a sampling period $T_{e}$ and these data are collected with *N* samples over one period $T_{e}$. We denote the *k*th sampling time as *t_k_*. These measurements can be used to obtain an over-determined set of equations [36]:

(25) $$\Gamma_{t}\left(\tau \right)=W(q,\hat{\dot{q}},\hat{\ddot{q}})\theta +\rho$$

where $q,\hat{\dot{q}}$ and $\hat{\ddot{q}}$ are vectors of joint positions, estimated velocities, and estimated accelerations, respectively. $\Gamma_{t}\in {\mathbb{R}}^{\left(n\times 1\right)}$ and $\rho \in {\mathbb{R}}^{\left(n\times 1\right)}$ are vectors that represent a sampling of the actual torques τ and all terms caused by the modeling error, friction, and measurement noise, respectively. *n* is the number of equations and $n_{\theta }$ is the number of parameters to be identified. ρ is supposed to have a zero mean and is serially uncorrelated. $W\in {\mathbb{R}}^{\left(n\times n_{\theta }\right)}$ is the observation matrix, which is a sampling of the regression *H* defined by (3). Then, $\Gamma_{t}$ and W are defined as follows:

$$\Gamma_{t}={\left[\Gamma_{t}^{1}\ldots \;\Gamma_{t}^{n}\right]}^{T};$$

where: $\Gamma_{t}^{j}={\left[\tau_{j}\left(1\right)\ldots \;\tau_{j}\left(N\right)\right]}^{T};$ $W^{j}=\left[\begin{array}{c} H^{j}(q\left(1\right),\hat{\dot{q}}\left(1\right),\hat{\ddot{q}}\left(1\right))\\ H^{j}(q\left(2\right),\hat{\dot{q}}\left(2\right),\hat{\ddot{q}}(2))\\ .\\ .\\ H^{j}(q\left(N\right),\hat{\dot{q}}\left(N\right),\hat{\ddot{q}}\left(N\right)) \end{array}\right]$, where $\Gamma_{t}^{j}$ and $W^{j}$ are the *N* equations of a subsystem *j* with *N* number of rows. $H^{j}(q\left(.\right),\hat{\dot{q}}\left(.\right),\hat{\ddot{q}}\left(.\right))$ is the *j*th row of the $\left(n\times n_{\theta }\right)$ matrix of the regressor given by Equation (3).

Finally, the over-determined set of the equation system (25) is solved using the RLS estimator with a constant forgetting factor.

Noise will limit the accuracy of parameters obtained by least squares and also the convergence rate of the RLS algorithm. To overcome such problems, the trajectory used in the identification process must be correctly chosen, which is called a persistently exciting trajectory [45]. The parameter identification results rely on the well-conditioned reduced observation matrix and therefore to obtain a unique LS solution. The unicity of this solution directly depends on the rank of the observation matrix W. The rank loss of this matrix may occur in two cases: (i) where there is a structural issue of the parameter identifiability problem; (ii) where there is a data deficiency due to a lack of consideration of the sufficiency of trajectory excitation. To obtain such a trajectory, two methods are usually used: (i) compute the trajectory based on some optimization criteria [45]; (ii) use special test movements done sequentially to excite each time some parameters. This special test consists of locking some joints while moving others. For our case, the generation of such test moves has been carried out, which is why the observation matrix is assumed to be a full-rank and well-conditioned matrix. For the identification experiment, the solution of Equation (25) may induce some bias, essentially due to the measurement noises. Therefore, it is better to use data filtering to improve parameter estimations by the RLS method.

## 3. System Application and Implementation of the Identification Procedure

### 3.1. Application System: Robot SCARA

**Figure 2:**
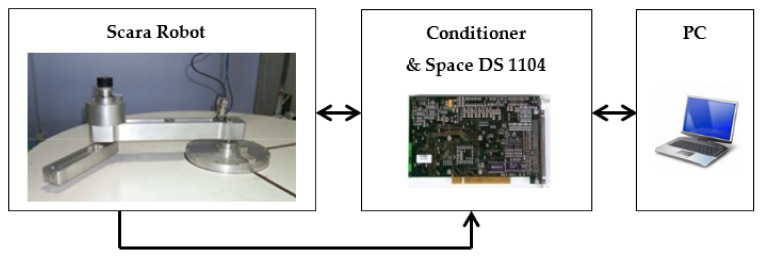
System application: Robot SCARA with 2-DoF.

The proposed identification scheme has been experimentally tested on the robot SCARA with 2-DoF without gravity and joints driven by synchronous motors with an absolute encoder (see Figure 2). The control law of the robot is validated via a Dspace 1104 controller board with a dedicated digital signal processor. The different terms defined in Equation (4) can be described as follows [17], where the regression matrix is given by:

(26) $$H(q,\dot{q},\ddot{q})=\left(\begin{array}{cc} {\ddot{q}}_{1} & 0\\ {\dot{q}}_{1} & 0\\ sign({\dot{q}}_{1}) & 0\\ {\ddot{q}}_{1}+{\ddot{q}}_{2} & {\ddot{q}}_{1}+{\ddot{q}}_{2}\\ (2{\ddot{q}}_{1}+{\ddot{q}}_{2})C_{O2}-{\dot{q}}_{2}(2{\dot{q}}_{1}+{\dot{q}}_{2})S_{O2} & {\ddot{q}}_{1}C_{O2}-{\dot{q}}^{2}{}_{1}S_{O2}\\ -(2{\ddot{q}}_{1}+{\ddot{q}}_{2})S_{O2}-{\dot{q}}_{2}(2{\dot{q}}_{1}+{\dot{q}}_{2})C_{O2} & {\dot{q}}^{2}{}_{1}C_{O2}-{\ddot{q}}_{1}S_{O2}\\ 0 & {\dot{q}}_{2}\\ 0 & sign({\dot{q}}_{2}) \end{array}\right)$$

with $C_{O2}=cos\left(q_{2}\right)$ and $S_{O2}=sin\left(q_{2}\right)$. The vector of unknown parameters’ vector is given by:

(27) $$\theta ={\left[ZZR_{1}\;F_{\upsilon 1}\;F_{s1}\;ZZ_{2}\;L_{1}MX_{2}\;L_{1}MY_{2}\;F_{\upsilon 2}\;F_{s2}\right]}^{T}$$

where $ZZR_{1}=ZZ_{1}+M_{2}L_{1}^{2}$ $L_{1}$ is the length of body 1, $M_{2}$ is the mass of body 2, and $ZZ_{i}$ represents the inertia’s moments of body *i*, $i=\left(1,2\right).$ $L_{1}MX_{2}$ and $L_{1}MY_{2}$ are the first moments of body 2 multiplied by the length of body 1, and $F_{\upsilon 1}$, $F_{s1}$, $F_{\upsilon 2}$, and $F_{s2}$ are the viscous and dry friction parameters of both axes.

### 3.2. Implementation of the Identification Procedure

The different steps required to implement the identification procedure are summarized as follows. (1) Make an adequate choice of the reference trajectory that must satisfy the persistency of excitation (PE) condition [44]. (2) Apply a controller for the system defined by Equations (4), (26), and (27). (3) Estimate both the velocity and acceleration for each joint with the proposed algorithms (7)–(8) and (9). (4) Inject the estimate signals into the regression matrix *W.* (5) Initialize the parameter vector (27) and the covariance matrix $P_{j},j\in \left\{0,\ldots ,N-1\right\}$. (6) Get the values of $q,\hat{\dot{q}},\hat{\ddot{q}},\Gamma_{t},{\hat{\Gamma }}_{t}$ at the time instant *nTe.* (7) Compute the *n*th estimate via $\hat{\theta }\left(n\right)$, (8) Update the parameters of the estimation model. (9)Go to step 6.

As indicated previously, the identification procedure is applied when the system operates in a closed-loop due to its instability in an open loop. Closed-loop identification is operated with a PD feedback control as the system tracks a fifth-order polynomial trajectory. The sampling frequency of the recording experimental data is equal to 200 Hz.

**Excitation trajectory.** The reference trajectory is computed to have a well-conditioned observation matrix *W* [45]. To satisfy the PE condition having an excitation trajectory $q_{ref}$, some conditions are imposed to define the polynomial trajectory as:

(28) $$\left\{\begin{array}{l} \left|q_{i}\left(t\right)\right|\leq q_{i\max};\left|{\dot{q}}_{i}\left(t\right)\right|\leq {\dot{q}}_{i\max};\left|{\ddot{q}}_{i}\left(t\right)\right|\leq {\ddot{q}}_{i\max}\\ q_{i}\left(t=0\right)=q_{i}\left(t=t_{final}\right)=0\\ {\dot{q}}_{i}\left(t=0\right)={\dot{q}}_{i}\left(t=t_{final}\right)=0\\ {\ddot{q}}_{i}\left(t=0\right)={\ddot{q}}_{i}\left(t=t_{final}\right)=0 \end{array}\right.i=\left\{1,2\right\}$$

where $q_{\max},{\dot{q}}_{\max}$, and ${\ddot{q}}_{\max}$ are bounds on joint positions, velocities, and accelerations, respectively. As shown in Equation (28), the values at the start (*t* = 0) and endpoints *(t = tf)* are null.

**Data filtering.** The PD controller is given by the following equation:

(29) $$\begin{array}{l} \vec{\tau }=K_{pr}{\vec{e}}_{q}+K_{dr}{\vec{e}}_{\dot{q}}\\ \left(\begin{array}{l} \tau_{1}\\ \tau_{2} \end{array}\right)=\left(\begin{array}{cc} K_{pr_{1}} & 0\\ 0 & K_{pr_{2}} \end{array}\right)\left(\begin{array}{l} \left(q_{1}-q_{1ref}\right)\\ \left(q_{2}-q_{2ref}\right) \end{array}\right)+\left(\begin{array}{cc} K_{dr_{1}} & 0\\ 0 & K_{dr_{2}} \end{array}\right)\left(\begin{array}{l} \left({\hat{\dot{q}}}_{1}-{\dot{q}}_{1ref}\right)\\ \left({\hat{\dot{q}}}_{2}-{\dot{q}}_{2ref}\right) \end{array}\right) \end{array}$$

where *K_pr_* and *K_dr_* are positive definite diagonal matrices for the proportional and derivative actions, respectively. It is worth noting that the PD controller has no impact on the accuracy of the parameter identification because such a control scheme allows for a very small error.

**Figure 3:**
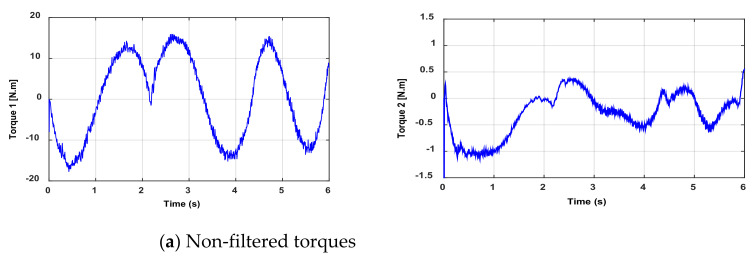

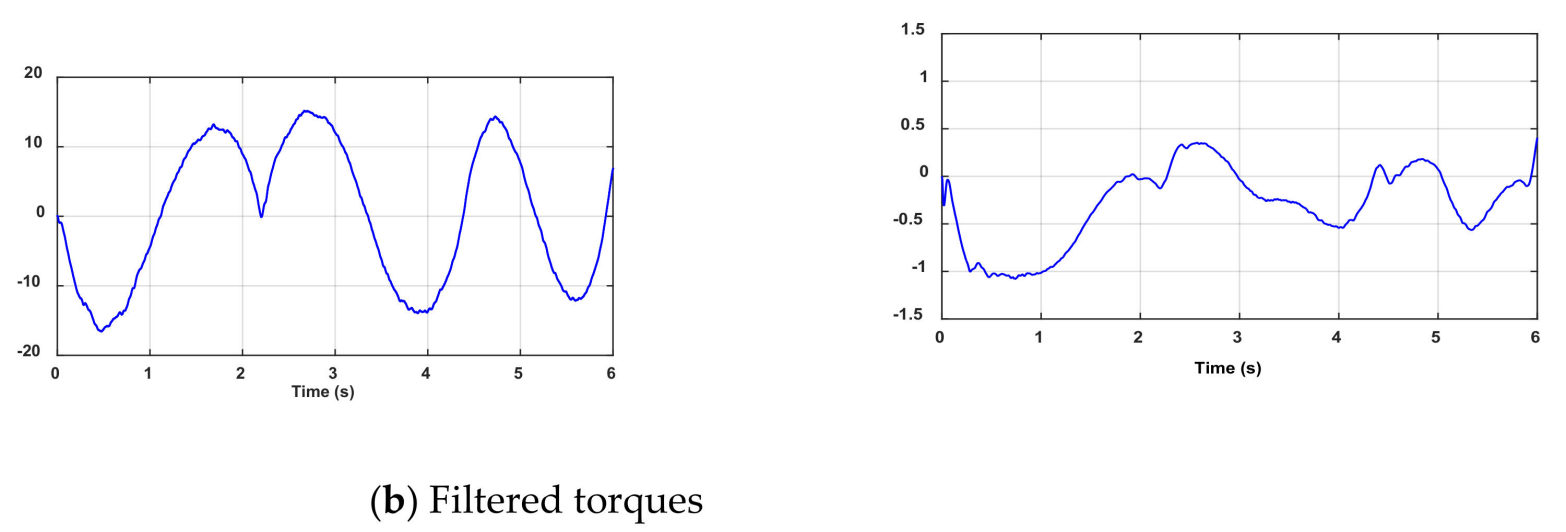
Curves of non-filtered and filtered torques.

The measurements are performed on the test bench in order to collect both the joint position and torque data. However, these data may be noisy and biased due to bad sensors, such as the quantization noise of the encoder. Then, to improve the accuracy of the parameter identification, a low-pass filter, e.g., the Butterworth filter, is used to treat data (inputs/outputs) online. This filter’s cutoff frequency $f_{F}$ is set to $f_{F}\geq 10f_{dyn}$, where $f_{dyn}$ represents the estimated natural frequency of the robot. The filtered torques are shown in Figure 3. All filters are implemented in their discreet form with the same sampling period of the control loop.

## 4. Experimental Results and Discussion

Different algorithms will also be exposed in order to consider them as a comparative basis to evaluate the proposed identification approach. Direct and cross-validations are performed, and the results are discussed, with some criteria of performance metrics analyzed to show the role of the soft sensor-based differentiator used in the parametric identification loop. Therefore, our aim is to compare the practical parameter estimation by the proposed differentiator P2SMD with the estimations by the classic sliding mode differentiator defined in Equation (4) (2SMD), the basic Euler differentiator (backward difference algorithm) associated with the FIR (finite impulse response) low-pass filter (ED+FIR), and a new scheme of the adaptive super twisting differentiator (ASTD) proposed by Shtessel [30]. This latter is described as follows:

(30) $$\left\{\begin{array}{l} {\dot{z}}_{0}=y_{0}\\ y_{0}=-k_{0}\left(t\right){\left|e_{0}\left(t\right)\right|}^{\frac{1}{2}}sign\left(e_{0}\left(t\right)\right)+z_{1}\;\; \end{array}\right.,\;\;\left\{\begin{array}{l} {\dot{z}}_{1}=y_{1}\\ y_{1}=-\frac{k_{1}\left(t\right)}{2}sign\left(e_{0}\left(t\right)\right)+z_{2} \end{array}\right.$$

The sliding function *e*_0_(*t*) is defined in the first equation of Equation (8). *k*_0_(*t*) and *k*_1_(*t*) are positive gains, where $k_{1}(t)=2\varepsilon k_{0}(t)$ and the dynamics of $k_{0}(t)$ are given by:

(31) $$\begin{array}{l} {\dot{k}}_{0}\left(t\right)=\left\{\begin{array}{ll} \alpha_{1}{\left(\frac{\mu_{1}}{2}\right)}^{\frac{1}{2}}sign\left(\left|e_{0}\left(t\right)-\gamma \right|\right) & if\;\;k_{0}>k_{0m}\\ \alpha_{0} & if\;\;k_{0}\leq k_{0m} \end{array}\right.\;,\\ \varepsilon ,\alpha_{1},\alpha_{0},\mu_{1},\gamma \;\mathrm{and}\;k_{0m}:\;\mathrm{positive}\;\mathrm{constants} \end{array}$$

For the experimental validation, an initialization step is necessary. For the RLS estimator, the initial covariance matrix $P_{0}$ is arbitrarily chosen such that it is a diagonal matrix. It is preferable that the values of the matrix coefficients are high in order to ensure fast convergence of the dynamic parameters. In practice, we have *P_0_* = 100*I*_1_, where *I*_1_ is an identity matrix. The values of the gains are set to $\alpha_{1}=0.95$ and $\alpha_{2}=1$. For the PD controller, its gain values are selected as *K_pr_* = [6.5 155]*^T^* and *K_dr_* = [5 25]*^T^*. The setting of each differentiation algorithm is described in Table 1.

**Figure 4:**
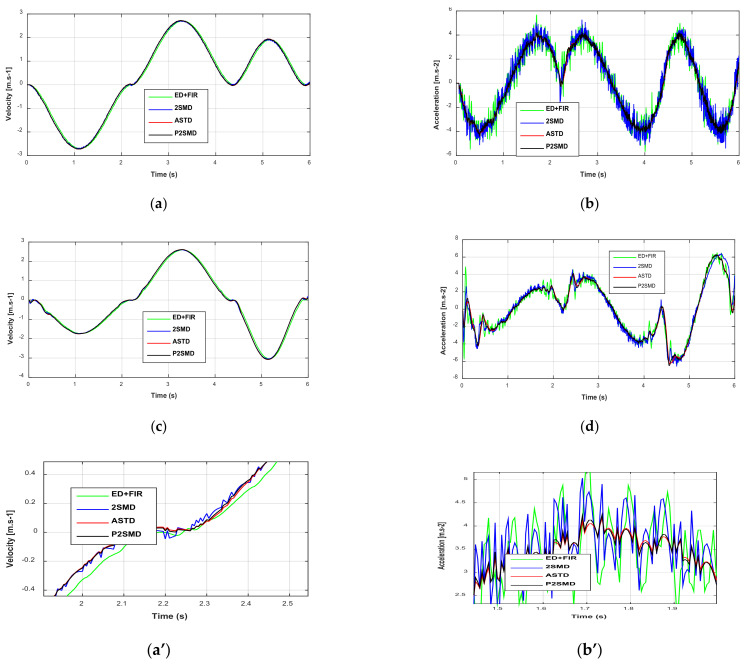

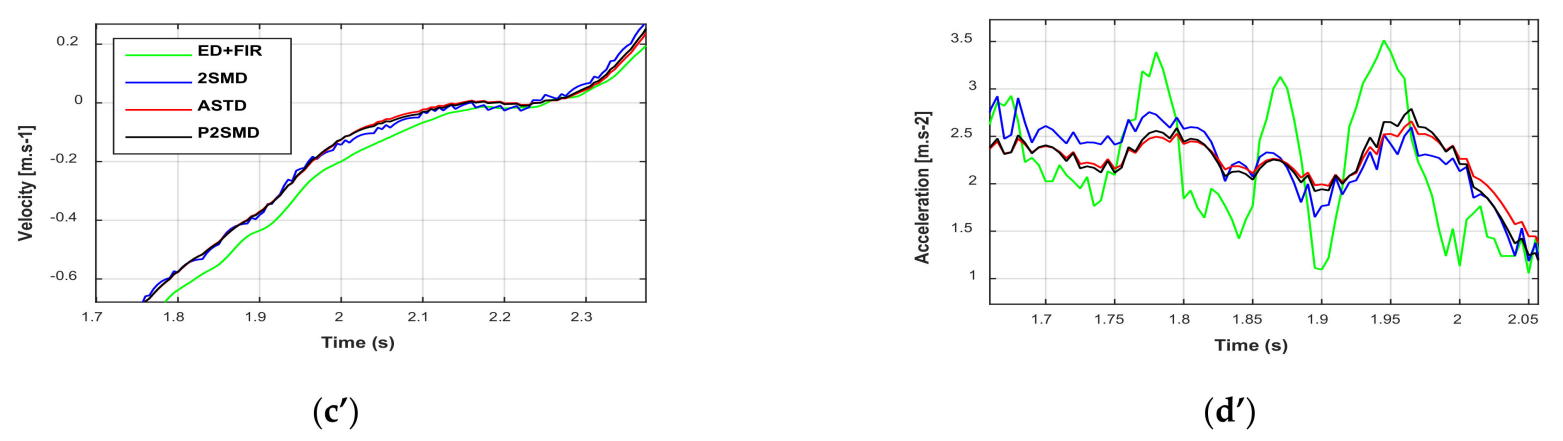
Estimated velocities and accelerations: (**a**) Time evolution velocities for joint 1; (**b**) Time evolution accelerations for joint 1; (**c**) Time evolution velocities for joint 2; (**d**) Time evolution accelerations for joint 2. Zoom of estimated velocities and accelerations: (**a’**) Time evolution velocities for joint 1; (**b’**) Time evolution accelerations for joint 1; (**c’**) Time evolution velocities for joint 2; (**d’**) Time evolution accelerations for joint 2.

**Figure 5:**
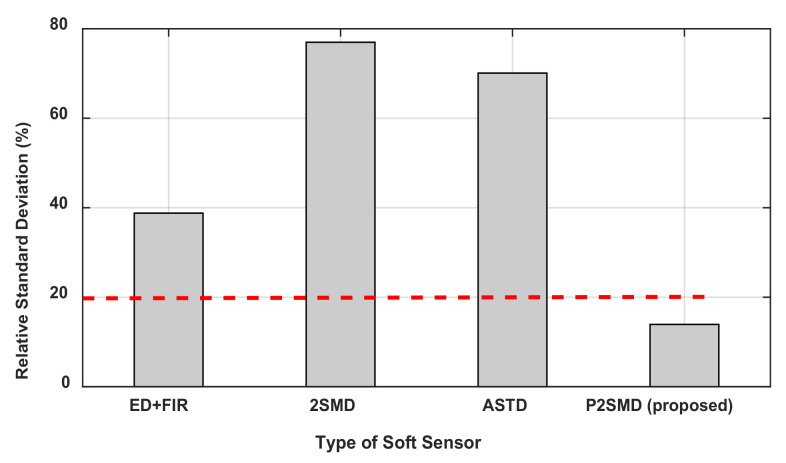
The maximum value of the relative standard deviation (%) for each differentiator.

It should be noted that the experiments were realized to measure the joint positions with an optical encoder. These position signals are affected by the quantization error. The velocities and accelerations estimated with the aforementioned differentiators are depicted in Figure 4 and Figure 5. We observe from Figure 4a and Figure 5a that the signals given for the velocities present low noise. On the other hand, the acceleration signals show amplification of the noise level (Figure 4b and Figure 5b). Moreover, the proposed algorithm presents the lowest noise level compared to the others. However, it must also be said that the signals estimated by the ASTD have a relatively long transient phase compared to the signals estimated by the other algorithms. In fact, if there is a change of setting to improve this transient phase, an increase in the level of the noise would then be recorded. Therefore, a delicate compromise exists with the adjustment of the ASTD.

### 4.1. Statistical Analysis and Comparison Criteria

To correctly compare the estimated parameters obtained by the different differentiators, let us define the relative standard deviations can determine assuming the matrix *W* to be a deterministic one. From Equation (25), ρ is a zero-mean additive independent noise such that:

(32) $$C_{\rho \rho }=E\left[\rho^{T}\rho \right]=I$$

where *I* is an *(n × n)* identity matrix. The covariance matrix of the estimation error is calculated by:

(33) $$C_{\hat{\theta }\hat{\theta }}=E\left[{\left(\theta -\hat{\theta }\right)}^{T}(\theta -\hat{\theta })\right]={\left(W^{T}W\right)}^{-1}$$

where $\sigma_{{\hat{\theta }}_{i}}^{2}=C_{\hat{\theta }{\hat{\theta }}_{ii}}$ is the *i*th diagonal coefficient of $C_{\hat{\theta }\hat{\theta }}.$ The relative standard deviation $\%\sigma_{{\hat{\theta }}_{i}}$ of ${\hat{\theta }}_{i}$ is given by:

(34) $$\%\sigma_{{\hat{\theta }}_{i}}=100\frac{\sigma_{{\hat{\theta }}_{i}}}{\left|{\hat{\theta }}_{i}\right|}\;\mathrm{with}\;\left|{\hat{\theta }}_{i}\right|\neq 0.$$

Focusing on other quantitative elements for the comparative study is also considered. A root-mean-square (RMS) error for each estimated parameter is computed as a criterion. This error is given by the following expression:

(35) $$RMS_{{\hat{\theta }}_{i}}=\sqrt{\frac{1}{N_{n}}\sum_{i=1}^{k}{\left(\theta_{i}-{\hat{\theta }}_{i}\right)}^{2}}$$

where *N_n_* is the number of samples and $\theta_{i},{\hat{\theta }}_{i},i=\left\{1,..8\right\}$ are the *i*th actual and estimated parameters, respectively. Good validation requires a good prediction of the torques of each joint. To quantify the quality of torque prediction, some criteria are calculated, such as the RMS error (RMSE) already defined in Equation (35), and the coefficient of determination, denoted by *R*^2^, is also computed to assess the strength of the linear relationship between the actual torques and the predicted ones. The formula used to calculate this coefficient is as follows:

(36) $$R^{2}=1-\frac{\sum_{i=1}^{N_{n}}{\left(\tau_{i}-{\hat{\tau }}_{i}\right)}^{2}}{\sum_{i=1}^{N_{n}}{\left(\tau_{i}-\frac{1}{N_{n}}\sum_{i=1}^{N_{n}}\tau_{i}\right)}^{2}}$$

where *N_n_* is the sample size and $\tau_{i},{\hat{\tau }}_{i},$ are the individual samples of the actual and the predicted torques, respectively, indexed with *i.*

### 4.2. Results of Direct Validation

Table 2 presents the parameters identified with the four algorithms as well as the a priori values of these parameters. The a priori values have been determined via measurements made on the disassembled links. From Table 2, one can notice that, of all the differentiation algorithms, the P2SMD has the lowest relative standard deviation for almost all the parameters. The deviations recorded for the frictions by the P2SMD are high but values remain lower than those given by the other algorithms. For example, for the dry friction $F_{v2}$, the value given by the proposed algorithm is 3.37 times lower than the ED+FIR value and more than 10 times lower than the ASTD value. In Figure 5, the maximum value $\%\sigma_{{\hat{\theta }}_{i}}$ is presented for each algorithm. It is clear from this figure that the lowest value is given by the proposed algorithm. For each of the other algorithms, the maximum value of the relative standard deviation greatly exceeds 30%.

If the values of $\%\sigma_{{\hat{\theta }}_{i}}$ exceed 20% or 30%, then the parameters are misidentified [8]. In this research work, the authors suggest that the parameters which remain poorly identifiable must be canceled because their contributions to the system dynamics are poor. However, there are no statistical tests that prove the cancelation or not of such parameters. However, we can conclude that the rather large $\%\sigma_{{\hat{\theta }}_{i}}$ proves that the algorithm used presents more noise and allows the estimator to be biased. Thus, one can observe that, except for the friction parameters ($F_{v1}$, $F_{s1}$, $F_{v2}$, $F_{s2}$), the obtained outcomes are widely accepted values of $\%\sigma_{{\hat{\theta }}_{i}}$. Therefore, it is more interesting to consider them as parameters varying over time, which can enhance their identification. Table 3 provides the results concerning the RMS error corresponding to each identified parameter. The lowest RMS error values are described in bold; these lowest values are given by the proposed differentiation algorithm.

**Figure 6:**
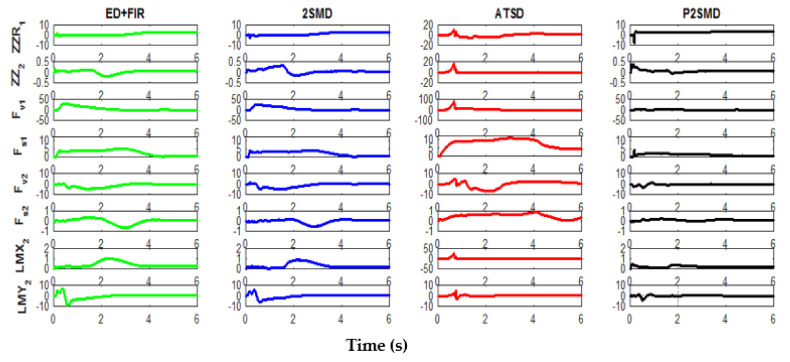
Time evolution of the estimated parameters using the different differentiators.

The time evolution of the estimated parameters using the studied and proposed algorithms is presented in Figure 6. Note from this figure that for the estimates provided by the ASTD, the time evolution of the parameters behaves in an oscillatory manner and has the highest transient phase. This adaptive form of the ST differentiator (ASTD) remains hard to adjust due to the large number of parameters required (six parameters) and the compromise that exists between the convergence speed, the noise level, and the accuracy of the algorithm. The evolutions of the estimated parameters obtained by the 2SMD and ED+FIR are quite close, although the 2SMD presents faster convergence with less overshoot for some parameters. With the proposed algorithm, the parameters are identified with the weakest transient phase and have the lowest overshoot. For some parameters, the estimated values converge to the a priori known values in 2 s.

**Figure 7:**
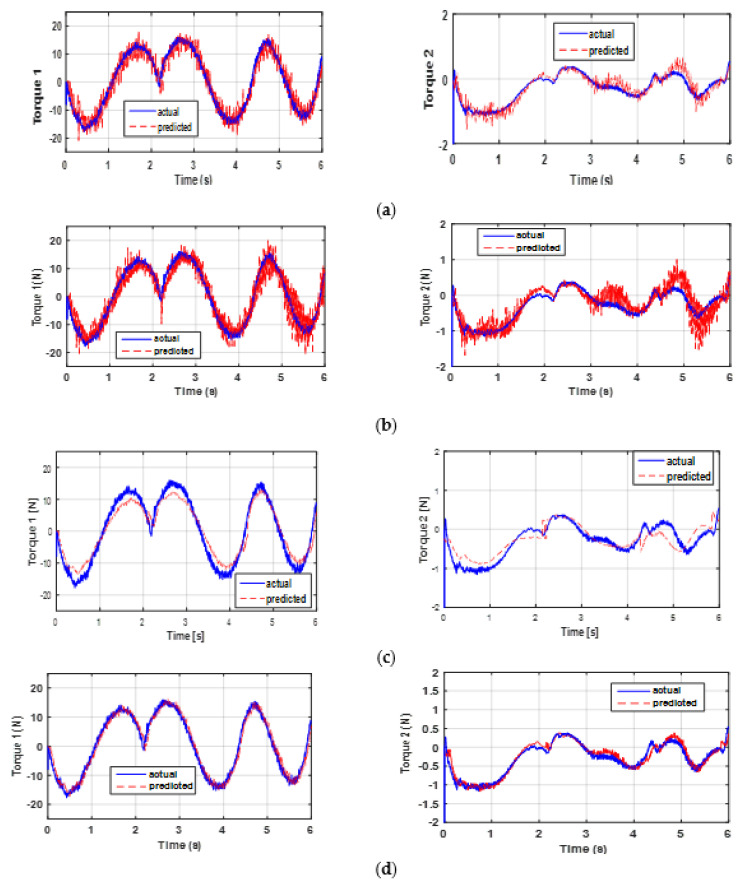
Actual and predicted torques: direct validation through (**a**) ED+FIR, (**b**) the 2SMD algorithm, (**c**) the ASTD algorithm, and (**d**) the P2SMD algorithm.

A direct validation is performed to test the model and to highlight its performance. The aim is to compare the actual torques with those estimated using the identified parameters (see Figure 7). Note that the predicted torques given by the ED+FIR are most affected by the noise (see Figure 7a). This is obviously because the velocity and acceleration estimates for this differentiator are much noisier than for the other differentiators. It is also worth mentioning that all disturbances, such as noise, position quantization error, and quality of velocity and acceleration estimations, remarkably affect the identification results.

Figure 7b,c show that the identified model and measurements are close with less noise than the ED+FIR. From Figure 7d, it is clear that the predicted torques are very close to the measured ones for the P2SMD. This implies a good estimation of the parameters. In addition, the proposed algorithm is easier to adjust than the other algorithms. As noted in Table 1, the P2SMD presents only two parameters that need adjustment. Table 4 shows the *RMS error* and the *R*^2^ values yielded by each differentiation method, with the best values highlighted in bold. In general, the validation gives better results for the prediction of torque 1 than for the prediction of torque 2. Indeed, for torque 1, the values of *R*^2^ and *RMSE* are quite close for the ED+FIR, 2SMD, and ASTD algorithms. The value of *R*^2^ for each of these three algorithms is about 91%, while the corresponding value for the proposed algorithm is 96%. The RMSE of the proposed algorithm is 1.5 times lower than those of the other algorithms. However, the results provided for torque 2 remain acceptable, with an *R*^2^ equal to 92%, 85%, 81%, and 72% for P2SMD, 2SMD, ED+FIR, and ASTD, respectively. Thus, the *R*^2^ values obtained with the proposed method are very close to 1 but the RMSE values are lowest for both torques, which indicates that the fit between the actual and predicted signals is almost perfect; therefore, the model is reliable. It is important to mention that the other differentiators give an acceptable value of *R*^2^ since they exceed 71%. However, the values of *RMSE* and *R*^2^ given by ASTD for torque 2 are the worst compared with other methods. Although this algorithm has a good filtering rate, its estimates are less accurate. For a given system, the set of parameter estimates could be valid for any inputs/outputs. Therefore, validation must be carried out with another input/output dataset to provide a conclusion on the quality of the parameter identification step or torque prediction step. The next section presents the result of the cross-validation of the model.

### 4.3. Results of the Cross Validation

For the cross-validation, we use the same parametric setting of the PD controller. The parametric setting of the different algorithms also remains the same. Other exciting trajectories different from those defined during the identification process are used.

**Figure 8:**
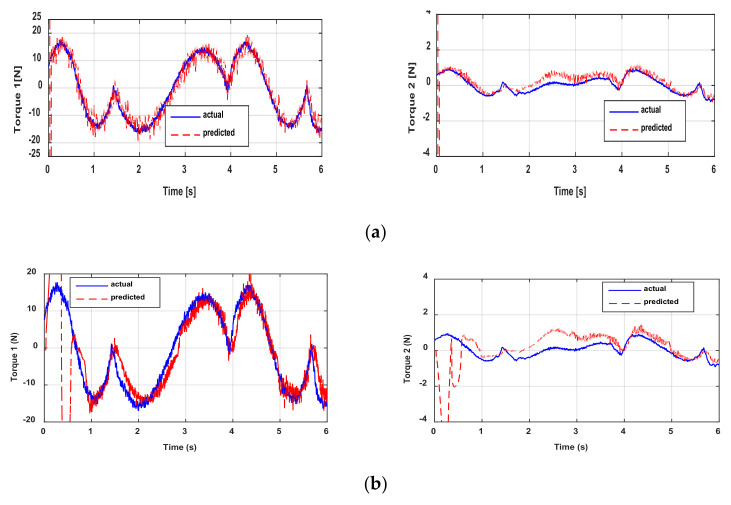

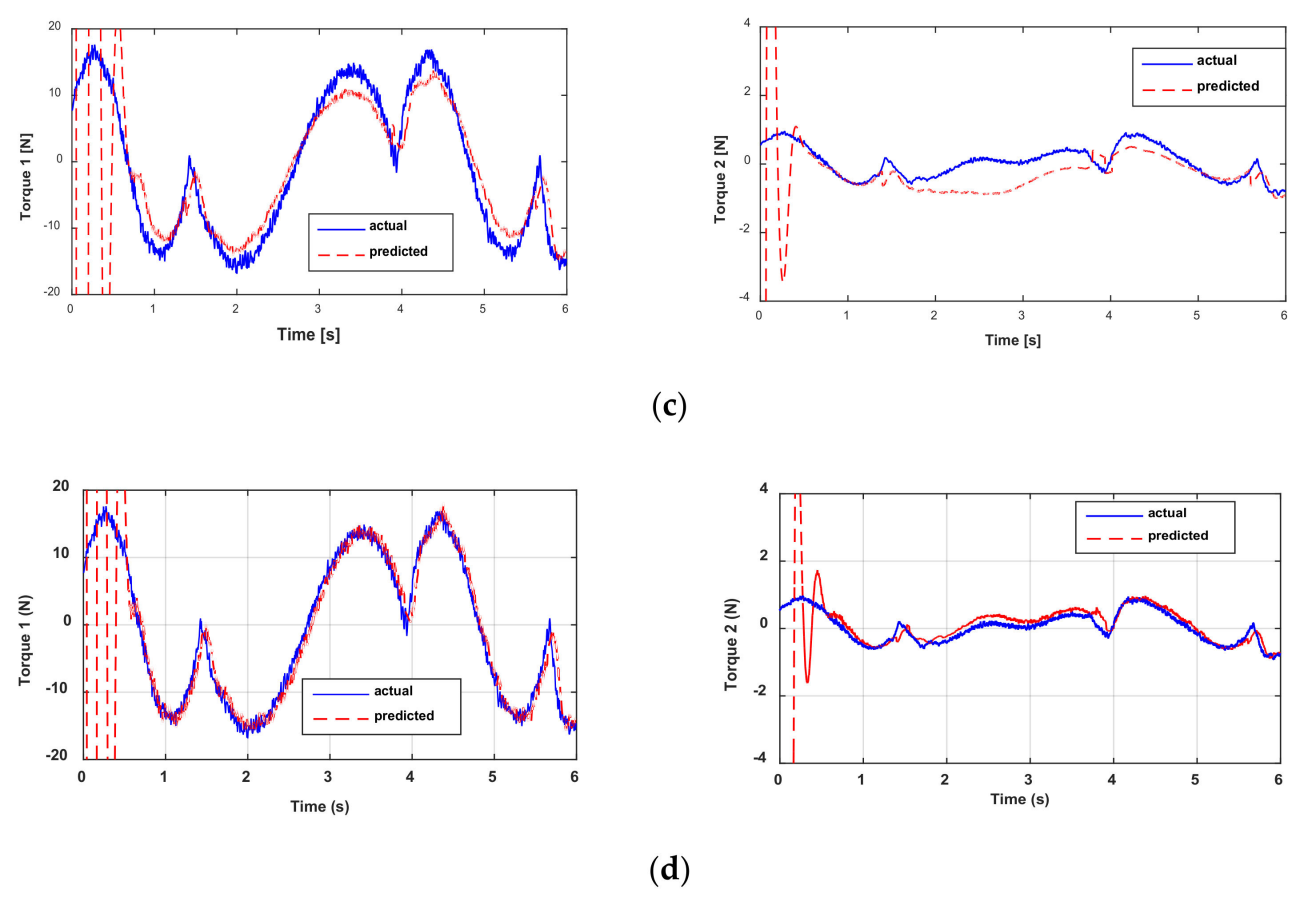
Actual and predicted torques: cross-validation through (**a**) ED+FIR, (**b**) the 2SMD algorithm, (**c**) the ASTD algorithm, and (**d**) the P2SMD algorithm.

Figure 8 shows the actual and predicted actuator torques. For torque 1, the cross-validation yields relatively good results. The results are not as good for torque 2 compared to torque 1. This is explained by the fact that the dynamics of the new trajectory of torque 2 is too different from that used for direct validation. Figure 8 indicates that the proposed method has the best performance and allows predicting the actuator torque with a good filtering rate. In general, algorithms based on sliding modes have some transient phases, but this remains weak, and all the algorithms quickly converge. It is possible to reduce this transient phase by increasing the gains of the algorithms. This is not a limitation for the proposed algorithm given that it presents a good level of filtering. The torque ripples can also be suppressed with a decimation procedure; this procedure is detailed in [22]. On the other hand, increasing the values of the gains tends to increase the noise level. Given the good filtering provided by the P2SMD, increasing the convergence gains will not pose such a challenge. Similar to the qualitative criterion for cross-validation via Figure 8, the quantitative criteria show good performance of the proposed algorithm. In fact, the P2SMD has the lowest values in terms of *RMSE* and the highest values for *R^2^,* see Table 5. Moreover, the *R*^2^ values are very close to 1, which shows that the model obtained makes it possible to correctly predict both torques. For torque 2, the results provided by the other algorithms are largely degraded especially for the ASTD. This is due to its high cumulative imprecision, since the estimation of acceleration is done via two successive blocks of ASTD.

## 5. Discussion

The parametric identification of a nonlinear model and the prediction of the inputs based on the inverse model associated with a differentiation algorithm have been studied in this paper. Such an approach can circumvent the non-convexity problem to find a local solution. In fact, this study describes the key role of a soft sensor for this purpose with a basic RLS algorithm. It is well known that the RLS method can provide unbiased results due to inaccurate measurements of joint positions *q* at a high sampling rate and especially due to the bad-tuned filtering. In the previous research works, one associates the RLS method with a traditional Euler type differentiator with a filter. The challenge of such an LS estimator concerns the noisy observation matrix; thus, the filter cut-off frequency must be correctly chosen. An alternative solution to the conventional differentiator associated with a filter is the soft sensors based on SM differentiators, in which the differentiation and filtering take place in a parallel manner. Two SM algorithms (2SMD and ASTD) have been compared to the proposed P2SMD. The results indicate a significant difference between the three studied algorithms (ED+FIR, 2SMD, and ASTD) and the P2SMD algorithm. The performance has been evaluated in terms of qualitative and quantitative criteria. The qualitative criteria are based on the quality of the curve forms of the predicted torques compared with the actual ones and on the time evolution of the estimated parameters given for all soft sensors. The quantitative criteria are defined by some performance metrics, such as the relative standard deviations, the coefficient of determination R^2^, and the RMSE. For all these performance criteria, the results show the higher performance of the proposed soft sensor, which may be attributed to the quality of both the velocity and acceleration estimates. In fact, the performance likely depends on three factors: time convergence, accuracy, and noise robustness of the differentiator. Despite the disturbances due to the position quantization error, the P2SMD algorithm has been found to behave faster and more accurately than the other sensors. Moreover, it has a high filtering rate compared to the other algorithms. Thus, the considered metric values almost do not change between the direct and cross-validations, which proves the effectiveness of the P2SMD. Furthermore, the gain adjustment of the proposed algorithm is easier to do compared with other methods, and it is also easy to implement. In fact, only two parameters are necessary for this adjustment compared to three for the 2SMD and six for the ASTD. The classic differentiator yields better results than the 2SMD and ASTD, but attention must be paid to its filter setting in order to avoid any distortion in the frequency range related to the closed-loop manipulator. In [7], the authors present an extended Kalman filter to identify the parameters of a 2-DoF manipulator such as the one used in this paper. With such a filter, it is not necessary to carry out any treatment on the measurement joint positions. However, the obtained results were very sensitive to the initial values. Therefore, the extended Kalman filter requires good a priori knowledge of the initial parameters and takes a very long computing time compared to the proposed algorithm. It is important to highlight that for all the algorithms, the performance may be increased by incorporating a variable forgetting factor (FF) to correctly track the parameter changes. Specifically, for the parameters of viscous and dry friction, it is possible to optimize their identification using a value of FF that is smaller than 1, since these variables are non-stationary parameters. In fact, friction models are generally nonlinear. Thus, it is possible to use the separable least squares as presented in [46], where the dynamic model is divided into two sub-models. The first sub-model contains all base parameters and the second one is a nonlinear model that defines the friction parameters.

Our study is mainly applied on the SCARA robot with 2 DoF and no gravity effect. As shown in [47], it will be very interesting to validate our algorithm on a more complex robot (six degrees of freedom and with gravity effect) with a considerable number of parameters.

## Figures and Tables

**Table 1:** Parametric tuning of algorithms.

Algorithm	Settings
ED+FIR	The cutoff frequency of the FIR low-pass filter is a 10th-order FIR tuned to 5 Hz
2SMD	$\eta_{0}=10,\eta_{1}=8,\eta_{2}=5$
ASTD	$\varepsilon =1,\;\;\alpha_{1}=50,\;\alpha_{0}=0.1,\;\mu_{1}=2,\gamma =1.5,\;k_{0m}=0.01$
P2SMD *(proposed)*	$\Gamma_{0}=25,\Gamma_{1}=20$

**Table 2:** Estimated parameters and the relative standard deviation values. High values are noted in bold.

$\theta_{i}$	*a Priori* Values	ED+FIR	$\%\sigma_{{\hat{\theta }}_{i}}$	2SMD	$\%\sigma_{{\hat{\theta }}_{i}}$	ASTD	$\%\sigma_{{\hat{\theta }}_{i}}$	P2SMD *Proposed*	$\%\sigma_{{\hat{\theta }}_{i}}$
**ZZ_1_R_1_**	3.42	3.13	1.01	3.02	0.82	2.90	0.88	3.34	0.28
**F_v1_**	0.07	0.255	**38.79**	0.50	**15.94**		**14.14**	0.08	**13.92**
**F_s1_**	0.58	0.45	**34.56**	0.16	**76.98**	1.67	7.10	0.59	7.29
**ZZ_2_**	0.064	0.058	1.44	0.061	1.25	0.05	2.82	0.063	0.81
**L_1_MX_2_**	0.131	0.169	1.02	0.15	1.00	0.03	9.50	0.14	0.72
**L_1_MY_2_**	0	0.038	4.33	0.031	4.88	−0.06	4.03	0.018	5.2
**F_v2_**	0.015	0.0156	**26.62**	0.04	9.27	0.0099	**70.09**	0.028	7.88
**F_s2_**	0.156	0.0583	11.40	0.05	**11.27**	0.23	4.80	0.0789	5.0

**Table 3:** RMS parameters errors. The lowest values are noted in bold.

	ZZ_1_R_1_	F_v1_	F_s1_	ZZ_2_	L_1_MX_2_	L_1_MY_2_	F_v2_	F_s2_
**ED+FIR**	1.3285	0.9907	1.5776	0.0627	0.2754	0.2203	0.7456	0.2356
**2SMD**	1.1709	0.8036	1.1709	0.0479	0.2127	0.1559	0.5724	0.1981
**ASTD**	2.1080	2.9089	5.3328	0.1763	0.5272	0.2034	1.8056	0.2949
**P2SMD** *(proposed)*	**0.2797**	**0.2291**	**0.3270**	**0.017**	**0.0703**	**0.0430**	**0.1683**	**0.0795**

**Table 4:** Results obtained for the direct validation in terms of *R*^2^ and *RMSE.* The best results are shown in bold.

	Torque 1		Torque 2	
	R^2^	RMSE	R^2^	RMSE
**ED+FIR**	0. 9122	2.9766	0. 8135	0.1770
**2SMD**	0.9140	2.9510	0.8519	0.1672
**ASTD**	0.9182	2.9018	0.7250	0.2207
**P2SMD** *(proposed)*	**0.9604**	**1.9776**	**0.9215**	**0.1093**

**Table 5:** Results obtained for cross-validation in terms of R^2^ and RMSE. The best results are shown in bold.

	Torque 1		Torque 2	
	R^2^	RMSE	R^2^	RMSE
**ED+FIR**	0.9099	2.8556	0.6014	0.2758
**2SMD**	0.8693	3.0320	0.5014	0.2976
**ASTD**	0.8934	4.0341	0.0888	0.4553
**P2SMD** *(proposed)*	**0.9552**	**2.2530**	**0.8537**	**0.1862**

## Data Availability

Data sharing not applicable.
